# The exon junction complex factor Y14 is dynamic in the nucleus of the beetle *Tribolium castaneum* during late oogenesis

**DOI:** 10.1186/s13039-017-0342-4

**Published:** 2017-11-09

**Authors:** Artem M. Kiselev, Irina S. Stepanova, Leonid S. Adonin, Florina M. Batalova, Vladimir N. Parfenov, Dmitry S. Bogolyubov, Olga I. Podgornaya

**Affiliations:** 1 Laboratory of Cell Morphology, Institute of Cytology, Russian Academy of Sciences, St. Petersburg, 194064 Russia; 2 Federal Almazov North-West Medical Research Centre, St. Petersburg, 197341 Russia; 3 ITMO University, Institute of Translational Medicine, St. Petersburg, 197101 Russia; 4 Department of Cytology and Histology, Faculty of Biology, St. Petersburg State University, St. Petersburg, 199034 Russia; 5 Far Eastern Federal University, School of Biomedicine, Vladivostok, 690950 Russia

**Keywords:** *Tribolium castaneum*, Y14 (Tsunagi), Oocyte nucleus, Karyosphere, Karyosphere capsule, Interchromatin granule clusters, Microinjections, Molecular cloning, Gene manipulation, Transcription in vitro

## Abstract

**Background:**

The oocyte chromosomes of the red flour beetle, *Tribolium castaneum*, are gathered into a knot, forming a karyosphere at the diplotene stage of meiotic prophase. Chromatin rearrangement, which is a characteristic feature of oocyte maturation, is well documented. The *T. castaneum* karyosphere is surrounded by a complex extrachromosomal structure termed the karyosphere capsule. The capsule contains the vast majority of oocyte RNA. We have previously shown using a BrUTP assay that oocyte chromosomes in *T. castaneum* maintain residual transcription up to the very end of oocyte maturation. Karyosphere transcription requires evidently not only transcription factors but also mRNA processing factors, including the components of the exon junction complex with its core component, the splicing factor Y14. We employed a gene engineering approach with injection of mRNA derived from the Myc-tagged Y14 plasmid-based construct in order to monitor the newly synthesized fusion protein in the oocyte nuclei.

**Results:**

Our preliminary data have been presented as a brief correspondence elsewhere. Here, we provide a full-length article including immunoelectron-microscopy localization data on Y14–Myc distribution in the nucleus of previtellogenic and vitellogenic oocytes. The injections of the fusion protein Y14–Myc mRNA into the oocytes showed a dynamic pattern of the protein distribution. At the previtellogenic stage, there are two main locations for the protein: SC35 domains (the analogues of interchromatin granule clusters or nuclear speckles) and the karyosphere capsule. At the vitellogenic stage, SC35 domains were devoid of labels, and Y14–Myc was found in the perichromatin region of the karyosphere, presumably at the places of residual transcription. We show that karyosphere formation is accompanied by the movement of a nuclear protein while the residual transcription occurs during genome inactivation.

**Conclusions:**

Our data indicate that the karyosphere capsule, being a destination site for a protein involved in mRNA splicing and export, is not only a specializes part of nuclear matrix separating the karyosphere from the products of chromosome activity, as believed previously, but represents a special nuclear compartment involved in the processes of gene expression in the case the karyosphere retains residual transcription activity.

**Electronic supplementary material:**

The online version of this article (10.1186/s13039-017-0342-4) contains supplementary material, which is available to authorized users.

## Background

The red flour beetle, *Tribolium castaneum*, a member of the family Tenebrionidae, is a significant pest wherever processed grain products are in abundance. *Tribolium* has a generation time of 3–8 weeks depending on temperature and can reproduce for a long time [1]. These features make *Tribolium* an ideal lab animal and it thus became an early and important model of classical genetics, in particular population genetics [2].

The *T. castaneum* genome is the first genome of a beetle that has been sequenced [3], and its database is being constantly revised to provide more comprehensive genomic information [4]. At an estimated 160 Mb, the euchromatic genome of *T. castaneum* is about one-third bigger than that of *Drosophila*. Also in terms of gene number, *T. castaneum* currently scores higher, with an estimated about 16 thousands genes [3, 5]. What is more noteworthy is that, by and large, *Tribolium* seems to be ‘less derived’ than some of other insects for which the genomes have been sequenced, in particular the fruitfly and the mosquito. Overall, *T. castaneum* shares more genes with humans than the dipterans do. *Tribolium* researchers are surely be tempted to use this finding to argue that this organism is ‘more ancestral’, ‘more representative’ or simply ‘less weird’ than *Drosophila* [2]. Now the beetle becomes a model of the developmental biology [6, 7]*.* The key event of development is gametogenesis, but morphological descriptions of the *T. castaneum* oocyte nucleus (germinal vesicle, GV) were absent in the literature until the beginning of our work [8].

In general, nuclear architecture comprises a number of extrachromosomal structures, nuclear compartments, or domains [9–11]. The great bulk of comprehensive studies that allow deciphering the role of nuclear domains in the support of mRNA during its journey in the nucleus to the nuclear pore are being made on mammalian tissue-culture somatic cells. It is still unclear whether the results of these studies are fully applicable for other cell types, especially for specialized cells such as the oocytes.

In many organisms including beetles, oocyte chromosomes join together in a limited volume of the GV, forming a karyosphere (karyosome) during the diplotene stage of meiotic prophase [12]. Oocyte chromosomes assembled into the karyosphere may be additionally separated from the rest of the nucleoplasm by a complex extrachromosomal structure termed the karyosphere capsule (KC). Similar KC develops in the nucleus of *Tribolium* oocytes. Biological significance of the KC is still unclear. It is generally assumed that the KC represents a specialized component of oocyte nuclear matrix supporting the chromosomes in large GVs [12, 13]. However, immunolocalization studies carried out on the GV of a weevil [14] and the red flour beetle [8] have suggested the KC could also represent a peculiar storage compartment for pre-mRNA processing factors. Our data shows that the perichromatin region (PR), i.e., the space between the KC and condensed chromatin, is rather the place of active residual transcription.

Nuclear speckles, or SC35 domains, also known as interchromatin granule clusters (IGCs) in terms of electron microscopy, are suggested as one of the most universal and evolutionarily conserved nuclear domains [15, 16]. They primarily represent nuclear sites to store pre-mRNA splicing factors [17–20], but extensive studies carried out during the last decades have introduced the idea that IGC functions are broader than those supposed previously, and these domains are involved in many other nuclear processes closely linked to gene expression [16, 21].

Nucleoplasmic transport and export of mRNA are important steps of gene expression, presenting an integral part of mRNA biogenesis [22]. It is now evident that the steps preceding export of mRNA-protein complexes (mRNPs) through nuclear pores make an essential contribution to the efficiency of mRNP export itself [23]. A number of RNA-binding proteins and/or protein complexes playing a role in RNA export bind mRNA before export, forming export-competent mRNPs. Some mRNA export factors are recruited to active genes during transcription [24], whereas other components of mRNA export complexes bind pre-mRNA later, in a splicing-dependent manner [25, 26].

Export of mRNPs is enhanced by evolutionarily conserved multiprotein transcription-export complex (TREX) that is deposited on the pre-mRNA near the 5′-end cap [27]. Also facilitating mRNP export is the exon-exon junction complex (EJC) that shares two proteins, Aly/REF and UAP56, with the TREX. The EJC consists of more than dozen proteins and comprises a constitutive core including proteins Y14–Magoh, eIF4A3 and MLN51 [28]. The EJC assembly in vivo occurs within or around nuclear speckles/IGCs [29, 30], and export-incompetent mRNAs which carry an incomplete or destabilized EJC are retained in the speckles [31].

In the present work, we studied nuclear distribution of the EJC core protein Y14 in the GV of *T. castaneum*. Our preliminary data have been presented as a brief one-page correspondence elsewhere [32]. Here, we provide a full-length article including immunoelectron-microscopy localization data on Y14–Myc distribution in the nucleus of previtellogenic and vitellogenic oocytes. A special attention was paid to possible relations between Y14 and oocyte nuclear domains: the karyosphere, its capsule and nuclear bodies. The plasmids encoding the fusion protein Y14–Myc were constructed. Myc-tagged Y14 mRNA synthesized in vitro was microinjected into the oocytes, and nuclear distribution of Y14–Myc was monitored with the use of immunofluorescent (IF) and immunoelectron microscopy (IEM) and antibodies against the Myc tag.

Nuclear distribution of the newly synthesized Y14–Myc was found to be quite dynamic. The fusion protein was localized in the PR of the karyosphere and in the KC. Y14 was also revealed in IGCs/speckles (SC35 domains) in the GV of previtellogenic but not vitellogenic oocytes. Localization sites of Y14–Myc in the PR of the karyosphere apparently correspond to the places of residual transcription. We suggest that the KC is the protein destination site, while oocyte nuclear bodies (IGCs/SC35 domains) represent transient domains for Y14.

## Methods

### Laboratory insects and tissue preparation

The wild-type strain of *Tribolium castaneum* (Herbst.) (Coleoptera–Polyphaga, Tenebrionidae) was reared at 28 °C on whole wheat flour containing 5% (*w*/w) brewer’s yeast which formed the upper substrate level, and the lower substrate of oatmeal. The larvae used for RNA extraction were kept for 1 day on dry filter paper at normal cultivation temperature. For IF and IEM studies, ovaries of adult females were isolated in the solution for insects (0.75% NaCl, 0.035% KCl, 0.021% CaCl_2_) or in Ex-Cell™ 420 insect medium (SAFC Biosciences) with 10% fetal bovine serum (Hyclone) [33].

### HEK293 cell line

The HEK293 cell line derived from primary human embryonic kidney cells, transformed with human adenovirus type 5 DNA and prepared with the use of standard techniques [34] was grown in Eagle’s minimum essential medium (MEM) supplemented with non-essential amino acids and ~10% calf serum. Transformation of the cells by Y14–Myc (pcDNA 3.1 Myc-His(A) + Y14 vector) was carried out using the calcium technique as described [35]. 3 days after exposure to DNA the cultures were switched to low Ca^2+^ medium [36] and examined periodically for the presence of the colonies of transformed cells. Then, the cells were grown routinely in Eagle’s medium containing 10% calf serum.

### Construction with unspliced *T. castaneum* Y14 gene

**Fig. 1:**
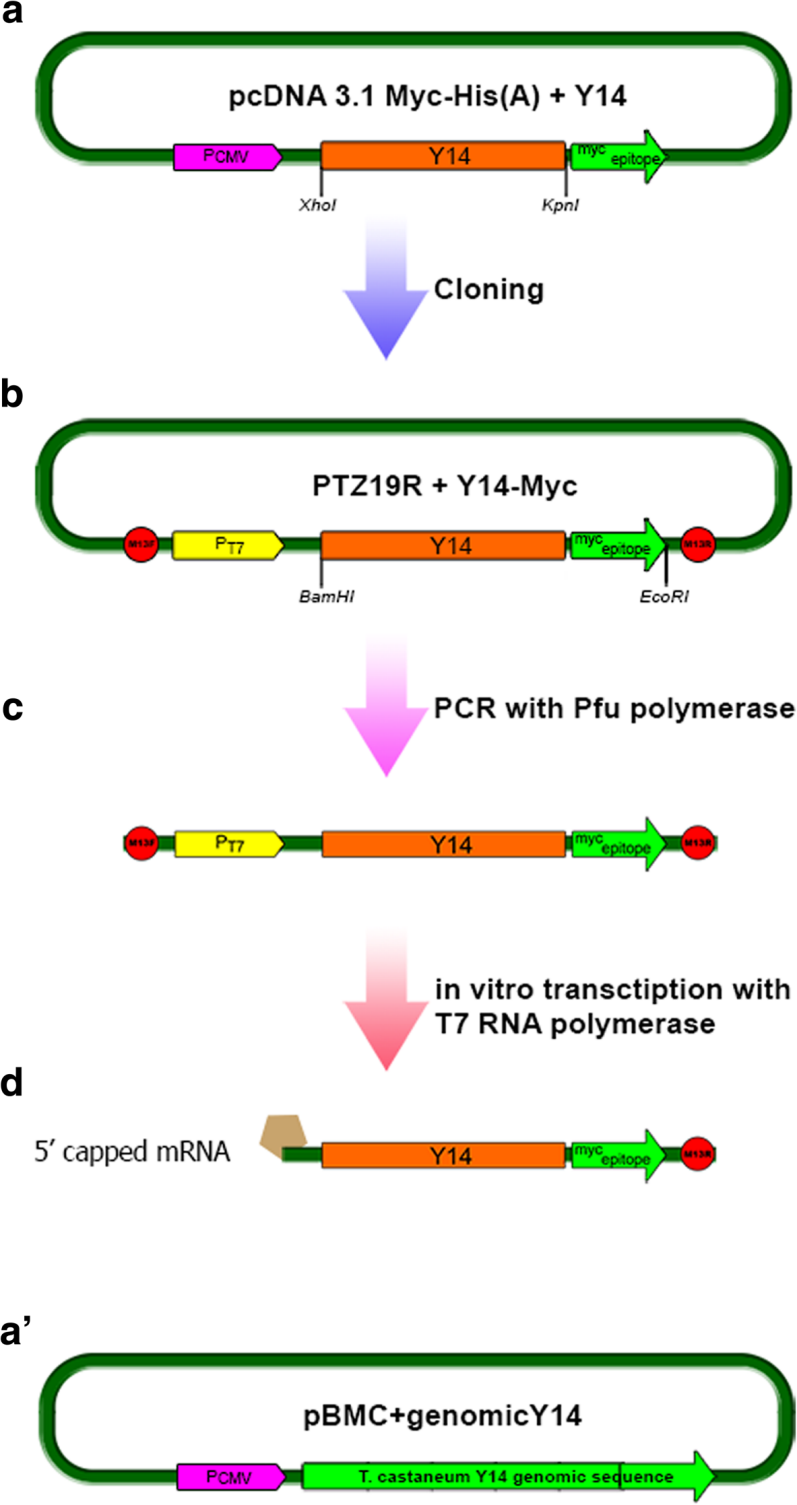
The scheme of Y14–Myc mRNA constructing (**a,b,c,d**) and Y14 genomic construction (**c**’). **a**, Y14 (506 bp) cloned in pcDNA 3.1 Myc–His; **b**, Y14 + Myc tag (542 bp) cloned in PTZ19R; **c**, PCR (639 bp) matrix for in vitro transcription; **d**, Y14–Myc mRNA (609 bp) transcribed in vitro. *Yellow*, T7 promoter; *orange*, Y14 coding sequence; *light green*, Myc epitope; *red circles*, M13 primers; *brown*, 5' cap analog; *dark green*, parts of the vector not significant in this case

The *Y14* sequence was selected from the *T. castaneum* genome DNA with the use of primers ## 7, 8 (Table 1; accession NCBI number: NW_001093646.1). The genomic *Y14* sequence was amplified, and then treated with appropriate restrictases (Table 1). The fragment was cloned in the pBMC vector containing the cytomegalovirus promoter (Fig. 1a’) [37]. Total RNA was extracted from cells 72 h after transfection, reverse transcription was performed, and the fragment of *T. castaneum Y14* gene was amplified with the same primers. The fragment was cloned and sequenced as described (see below), the result was a plasmid pBMC + genomicY14 (Fig. 1a’).

**Table 1:** List of primers

##	Primer Name	Sequence (5’–3’)	Inserted sites
1	006-XhoI-RNA-Forv	TTCTCGAGATGGCAG**ATG**TTTTGGACA	XhoI
2	007-KpnI-RNA-Rev	TAGGTACCGTGTCTCCTTGCACGTTTC	Kpn1
3	020-BamH1-RNA-Forv	GAGGATCCATGGCAGATGTTTTGGACA	BamH1
4	011-EcoRI-Stop-Myc-Rev	TCGAATTCTCAATTCAGATCCTCTTC***TGA***GATG	EcoR1, TGA(stop)
5	M13(-20) Forv	GTAAAACGACGGCCAGT	
6	M13(-26) Rev	GGAAACAGCTATGACCA	
7	001-DnaNheI-Forv	GTGCTAGCCACC**ATG**GCAGATGTTTTGGAC	NheI
8	002-DnaNot-Rev	ACGCGGCCGCTCAGTGTCTCCTTGCACG	NotI

Bold, start codon; bold italic, stop codon

### CDS Y14–Myc construction

Total mRNA was isolated from *T. castaneum* larvae using TRI-REAGENT (Sigma Aldrich) according to the standard acid phenol-guanidinium thiocyanate-chloroform extraction protocol. cDNA was prepared using RevertAid First Strand cDNA Synthesis Kit (Termo Scientific). For routine DNA amplification, Taq polymerase (Termo Scientific) was used according to standard recommendations [38].

Y14 sequence was obtained from NCBI (Gene ID: 656135). Specific primers were constructed, and the 5′ end was modified in order to insert restriction enzyme recognition sites (## 1, 2 in Table 1). These primers were used for routine DNA amplification with Taq polymerase (Termo Scientific) according to standard recommendations [38].

After PCR reaction, a 506 bp fragment (full length without stop codon) was amplified. The fragment was purified from gel using cut gel purification protocols (TermoScientific). Then, the fragment was ligated in PTZ57R/T TA cloning vector (Termo Scientific) according to standard InsTAclone PCR Cloning Kit protocol – TA cloning procedure. The resulting vector was transfected by the standard *E. coli* electroporation method [38]. The clones were examined by PCR. The plasmid was purified with QIAGEN–QIAprep Spin Miniprep Kit and restricted by XhoI and KpnI restriction enzymes (Termo Scientific). The fragment was cut from gel and cloned in pDNA 3.1 Myc-His(A) vector (Invitrogen) using the Myc tag and cytomegalovirus promoter (Fig. 1a).

The pairs of primers specific to the full length Y14*–*Myc (## 3,4 in Table 1) were used in a subsequent PCR to generate 506 bp full length *T. castaneum* Y14 + Myc with downstream stop codon in order to clone this fragment in PTZ19R vector (Termo Scientific) containing T7 promoter for in vitro transcription (Fig. 1b). Resulting plasmid B was checked for correct assembling with the correspondent pairs of primers by PCR.

**Fig. 2:**
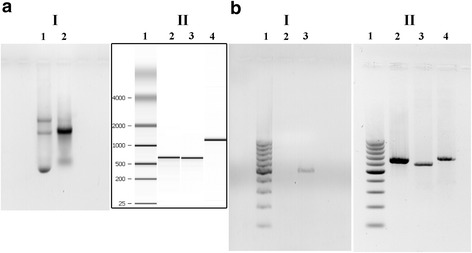
Electrophoretic control of the fragments of Y14–Myc construction. **a**, RNA electrophoresis. I — RNA electrophoresis under denaturing conditions; line 1, total RNA from rat liver (for control); line 2, total *T. castaneum* RNA from pupae. II — RNA transcribed in vitro and analyzed with microfluidics-based Agilent 2100 Bioanalyzer; line 1, a ladder (Agilent 2100 Bioanalyzer built-in function); lines 2 and 3, 5'-capped *T. castaneum* Y14-Myc RNA transcribed in vitro, 200 ng and 100 ng, respectively; line 4, mouse GABDH mRNA (for control, 200 ng). **b**, DNA electrophoresis. I — DNA electrophoresis of the reverse transcription PCR products; line 1, DNA ladder (100 bp); line 2, negative control; line 3, PCR with cDNA after RT-PCR with total *T. castaneum* RNA (506 bp), primers ## 1, 2 (see Table 1). II — DNA electrophoresis of PCR products amplified with Pfu polymerase on the PTZ19R + Y14-Myc vector for in vitro transcription (639 bp); line 1, DNA ladder (100 bp); line 2, primers ## 5, 4 (Table 1); line 3, primers ## 3, 6 (Table 1); line 4, primers ## 5, 6 (Table 1)

The plasmid A design (Fig. 1a) gives the possibility to transfect the cultured cells to check transcription and translation. The plasmid B with the inserted T7 promoter (Fig. 2b) was designed for in vitro transcription of the whole Y14–Myc mRNA.

### mRNA synthesis in vitro

The templates used for mRNA synthesis in vitro were PCR products prepared using Pfu polymerase for error-free amplification (Termo Scientific) and universal M13 forward and reverse primers (## 5,6 in Table 1) to amplify full length fusion Y14–Myc with the T7 promoter located upstream (Fig. 1c). RNA (Fig. 1d) was synthesized on the template using the TranscriptAid™ T7 High Yield Transcription Kit (Termo Scientific) with adding of Ribo m^7^G Cap Analog (Promega) for 5′ mRNA capping based on the standard TranscriptAid™ protocol. DNA templates and other reaction components were removed with RNAqueous kit (Ambion). RNA electrophoresis under denaturing conditions with PFA was performed according to the standard protocol [38] (Fig. 2 II, 2;3).

### Microinjections

Separate ovarioles, anatomical units of insect female gonads, were used for microinjections into the oocytes. The injections were carried out by using glass capillaries and an Eppendorf 5242 microinjector equipped with a Narishige micromanipulator. After microinjection, the ovarioles were kept in a moist chamber for 4–6 h, then squashed and prepared for IF or IEM (see below). Approximately 0.1 μl of 50 ng/μl mRNA solution (5 ng RNA per oocyte) was microinjected into the ooplasm [39]. About 30 oocytes were injected. Approximately 15% oocytes were survived the procedure and used for immunostaining experiments.

### Immunofluorescent microscopy

For IF microscopy, squashed preparations of oocyte nuclei were prepared as described [40, 41]. Ovarioles were gently squashed on a siliconized coverslip with a Polysine® microscope slide (Menzel) and frozen in liquid nitrogen. After the coverslip had been removed with a razor blade, the slide was fixed in 2% formaldehyde freshly prepared from paraformaldehyde (Ted Pella) in 96% ethanol for 45 min, rinsed in 70% ethanol and finally in PBS.

Preparations were incubated in 10% fetal calf serum (Gibco) in PBS for 10 min to prevent non-specific antibody binding. The incubation in primary antibody solution was carried out overnight in a moist chamber at 4 °C. After rinsing in PBS, preparations were incubated with secondary antibodies conjugated with Alexa-488 or Alexa-568 (Invitrogen, dilution 1:200) for 1.5 h at room temperature. To visualize DNA, preparations were additionally stained with 1 μg/ml To-Pro-3 dye (Molecular Probes) for 5 min, then rinsed in PBS and mounted in Vectashield® medium (Vector Laboratories) or directly mounted in the medium containing 1 μg/ml DAPI. For simultaneous DNA and RNA visualization, acridine orange staining method [42] was used*.* The samples were examined with a Leica TCS SP5 confocal laser scanning microscope equipped with Ar-UV diode (405 nm), Ar- (488 nm) and He–Ne- (543 and 633 nm) lasers. Confocal images were taken with a 63× objective (NA 1.32). Merged images were obtained using ImageJ 1.47.

### Immunogold labeling electron microscopy

For IEM, single ovarioles with injected oocytes were prefixed for 2 h in in PBS containing 4% formaldehyde and 0.5% glutaraldehyde at room temperature, and then fixed overnight in 2% formaldehyde at 4 °C. After rinsing in PBS containing 0.05 M NH_4_Cl and subsequent dehydration in an ethanol series, ovarioles were embedded in LR White resin (medium grade, Sigma). Ultrathin sections were incubated for 10 min in blocking buffer containing 0.5% fish gelatin (Sigma) and 0.02% Tween-20 in PBS, pH 7.4. After blocking, the sections were incubated overnight in antibody 9E10 solution in a moist chamber at 4 °C. After rinsing in TBS-Tween (TBST), the sections were incubated in the solution of biotinylated goat anti-mouse antibodies (Vector Laboratories) diluted 1:50 in TBST for 1 h at room temperature. After rinsing in TBST, the sections were incubated in the solution of gold-conjugated (15 nm) streptavidin (Aurion) diluted 1:20 in TBST. The sections were contrasted with uranyl acetate and examined in a Libra 120 electron microscope at 80 kV.

### Primary antibodies

Primary antibodies used were the following: goat polyclonal antibody C-20 raised against a peptide at the C-terminus of human Y14 (Santa Cruz Biotechnology, Inc.) (IF, dilution 1:50), rabbit polyclonal antibody ab9106 (Abcam) (IF, dilution 1:100) or mouse monoclonal antibody 9E10 against Myc epitope [43] (IEM, culture supernatant), mouse monoclonal antibody against SC35 [44] (IF, 2.5 μg/ml), mouse monoclonal antibody 030 against double-stranded DNA (Chemicon) (IEM, dilution 1:300).

### Computer based methods

Ugene software and Jalview software were used for the initial search of Y14 in the BeetleBase. Cloning in silico was made with Geneious and Bioedit tools. Primers were selected using Primer 3 plus web-based tool. Protein alignment was made by the Geneious software. The sequence of *Y14* gene was obtained from the NCBI database (Gene ID: 656135).

## Results

### *T. castaneum* Y14 gene

We took the advantage of the wealth of background genetic information with a recently published beetle draft genome [3]. The Y14 proteins extracted from the databases indicated were aligned (Additional file 1: Figure S1). The preliminary analysis shows that *Tribolium* shares more genes with humans than the dipterans do [2], and Y14 alignment confirms this. The alignment showed a high degree of homology, and all the main human protein features are present in the *T. castaneum* protein. Human and beetle Y14 mRNA sequences are similar in general, especially in RRM domain region, but in a NLS/YNS region there is less similarity. The direct (non-degenerate) primers have been designed from the sequence to pick up the beetle gene (Table 1).

### Y14–Myc construct

The construct was designed for accurate detection of Y14 pathways and its localization in the *T. castaneum* GV [32]. The Myc epitope was chosen as a tag due to its low impact on the structure of the fusion protein. Two constructions were made. The first construct was used to add the Myc epitope sequence to the Y14 coding sequence and for the control of its transcription activity in HEK293 cells (Fig. 1a). The second construct was used for transcription in vitro (Fig. 1b). The principal scheme of the constructing is shown in Fig. 1 (see also Materials and Methods).

Although the strategies are straightforward, a care should be taken to design plasmid constructions that encode protein tags, to generate high-quality mRNA in an RNase-free environment and to control the nonspecific effects of exogenous mRNA. In most vectors, protein expression is driven by the enhancer/promoter cassette of the immediate early gene of human cytomegalovirus. This cassette generates high levels of protein expression in most mammalian cell lines and is suitable for in vitro expression. The human cytomegalovirus major immediate early promoter is commercially available for the expression of various heterologous genes.

### Plasmid transcription and Y14 mRNA splicing in human HEK293 cells

**Fig. 3:**
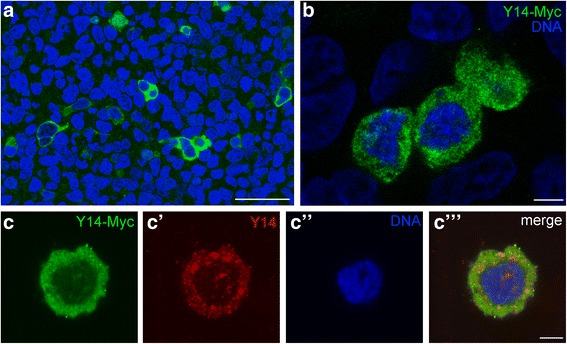
Plasmid A expression in HEK293 cells transfected with Myc-His-A with inserted Y14 sequence. Transfected cells at low (**a**) and high (**b**) magnification; anti-Myc staining is *green*, DNA stained with DAPI is *blue*. **c**—**c**″’, a transfected cell stained with antibodies against the Myc epitope (*green*) and Y14 (*red*); the nucleus is counterstained with DAPI (*blue*). Bars represent 50 μm in A and 5 μm in **b**, **c**—**c**″’

Prior to Y14–Myc RNA microinjections into *T. castaneum* oocytes we tested the transcription activity of *T. castaneum* Y14 coding sequence in HEK293 cells. The best way to check whether a plasmid was constructed correctly, is obtaining its transcription in the transfected beetle cell line, but such a line is not established yet. So, the human HEK293 cells were transfected with the plasmid A by calcium method. No less than 10% cells were transfected and expressed fused Y14–Myc (Fig. 3a, b). The fusion protein was localized mainly in the cytoplasm rather than in the nucleus (Fig. 3b). Such a behavior might be explained by the differences of NLS in human and beetle genes (Additional file 1: Figure S1).

We also checked whether *T. castaneum* Y14 pre-mRNA splicing is correct in human cells. The sequencing of 10 clones has shown that all the sequences are identical and differ from normally spliced Y14 mRNA (Additional file 2: Figure S2.). The *T. castaneum* Y14 pre-mRNA being processed in HEK293 cells does not lose the first intron (Additional file 2: Figure S2); two redundant GC nucleotides remain at the 3′ end of this intron. Exons 2 and 3 are being removed during splicing, while exon 4 remains. Thus, the plasmid is transcribed successfully, but is not being processed in the heterologous system. The reasons of this require a special study*.* The abortive splicing of insect pre-mRNA in human cells is in accordance with the data published [45].

Abortive splicing might explain why the location of the fusion protein and the intrinsic Y14 in HEK293 cells were found rather different after double immunostaining with antibodies against the Myc tag and Y14 (Fig. 3c–c”’). The bright foci corresponding Y14 in the vicinity of the nucleus probably represent translation factories that do contain the fusion protein. Nuclear intrinsic Y14 is visible in spots, which probably correspond to speckles. These domains do not include the fusion protein. The fusion protein seems cannot be imported to the nucleus of human cells due to unique NLS (Additional file 1: Figure S1). Nevertheless, the plasmid A is being transcribed successfully in human somatic cells. Still, to ensure the main aim of this study, we have constructed the plasmid B (Fig. 1) in order to synthesize in vitro 5′-capped mRNA and inject this RNA into the oocytes.

### Oocyte stages, karyosphere capsule morphology and the perichromatin region

**Fig. 4:**
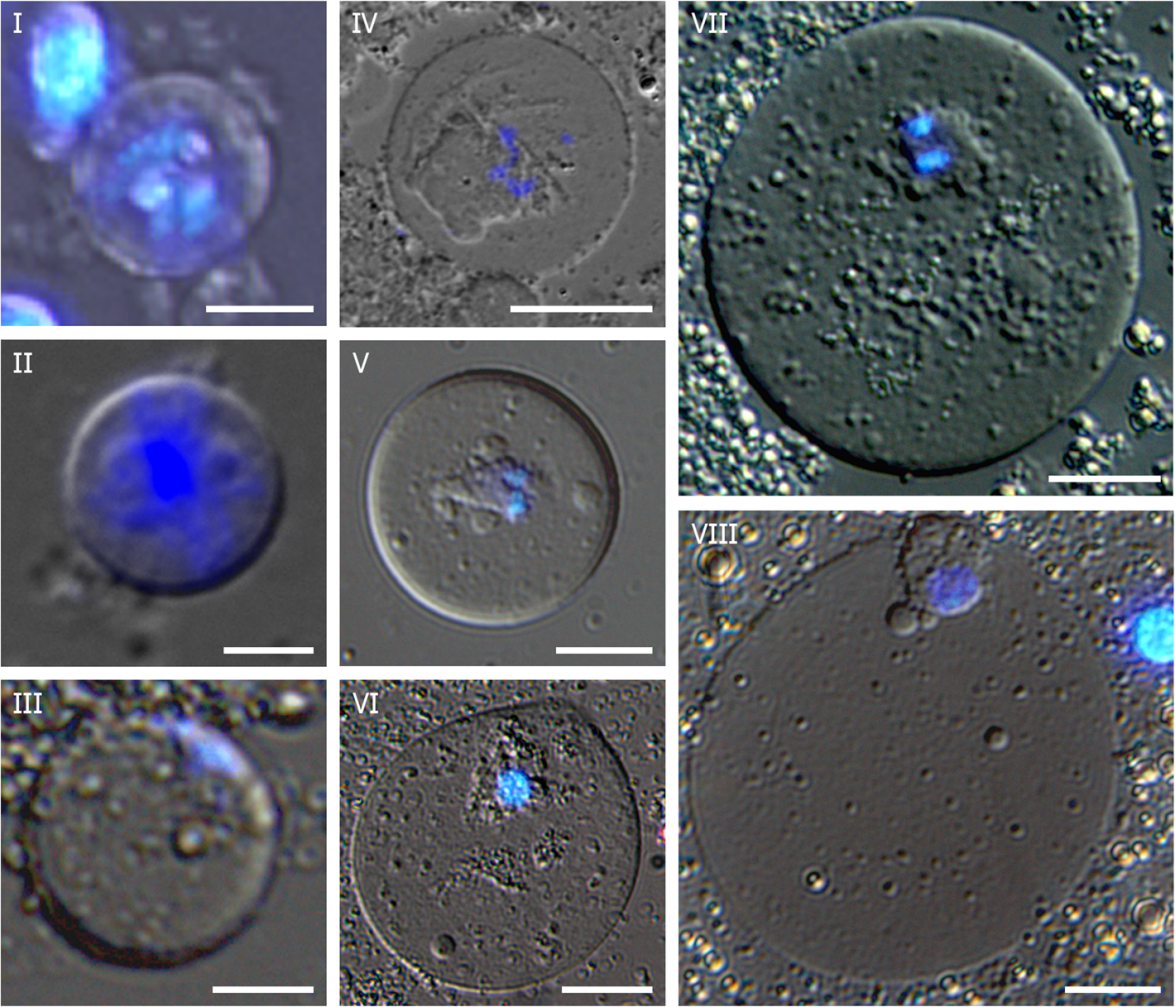
Morphology of the *T. castaneum* germinal vesicles at different stages of oocyte growth, squashed preparations of the stage **I** oocyte and the GVs from later stage oocytes. DIC, DAPI staining. Chromatin is dispersed and active transcription occurs at the stages **II**-**III**, before karyosphere formation that begins at the stage **IV**; **V** — late previtellogenic stage, a karyosphere capsule is formed; **VII** — vitellogenic stage, the capsule is fully developed, reaching its maximal complexity. Bars represent 5 μm for **I**—**II**; 10 μm for **II**; 20 μm for **III**—**VIII**

Initially, we characterized oocyte stages in *T. castaneum* adult females morphologically with special emphasis on the development of the karyosphere and its capsule (KC). *T. castaneum* oocytes develop in typical meroistic telotrophic ovaries. The imago ovaries contain already differentiated oocytes and nurse cells [46]. General morphology of follicles, follicular epithelium and the stages of vitellogenesis studied in *Tenebrio* [47] together with our observations on the morphodynamics of *Tribolium* oocyte nuclear structures including the karyosphere and KC allowed us to propose a nomenclature for the stages of *T. castaneum* oocyte development during the long-term diplotene [8]. In *T. castaneum* oocytes, karyosphere formation begins in the stage IV oocyte, and the KC reaches the highest morphological complexity at the stages V–VII. The stages of *T. castaneum* GVs based on morphological criteria are presented in Fig. 4.

**Fig. 5:**
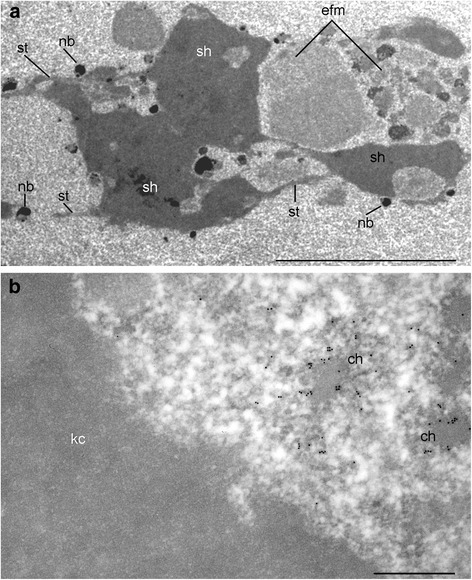
Ultrastructure of a fragment of *T. castaneum* karyosphere capsule in the stage 7 oocyte (**a**) and a fragment of karyosphere after immunogold labeling with anti-DNA antibody (**b**). In the karyosphere capsule, electron dense ‘the shell material’ (sh) and fibrillar ‘the strands’ (st) as well as the entangled filamentous material (efm) of medium electron density are distinguished; several nuclear bodies (nb) are located in the vicinity of the capsule (**a**). Condensed chromatin clumps (ch) and perichromatin region containing diffuse DNA loops are labeled with anti-DNA antibody; karyosphere capsule (kc) is devoid of the labels (**b**). Bars represent 5 μm in A and 0.5 μm in B

A well-developed KC in *T. castaneum* oocytes (Fig. 5a) was found to consist of three main different structures: (i) an electron-dense “shell” containing F-actin; (ii) fibrillar strands also containing actin and organized into cross-striated bundles; and (iii) lamin B-containing filamentous material consisting of entangled threads. Several nuclear bodies are scattered in the nucleoplasm and some are often seen attached to the KC. Amongst these bodies, coilin- and SC35-containing domains have been identified [8].

During the period of the existence of karyosphere, chromatin condensation is observed in the *T. castaneum* GV, but it is not absolute. Labeling of ultrathin sections of the *T. castaneum* GV with an anti-DNA antibody revealed compact chromatin blocks and loose DNA loops in the space abut to the KC (Fig. 5b). The latter space (the perichromatin region, PR) could be a place for residual transcription.

### RNA amount and residual transcription in the GV

**Fig. 6:**
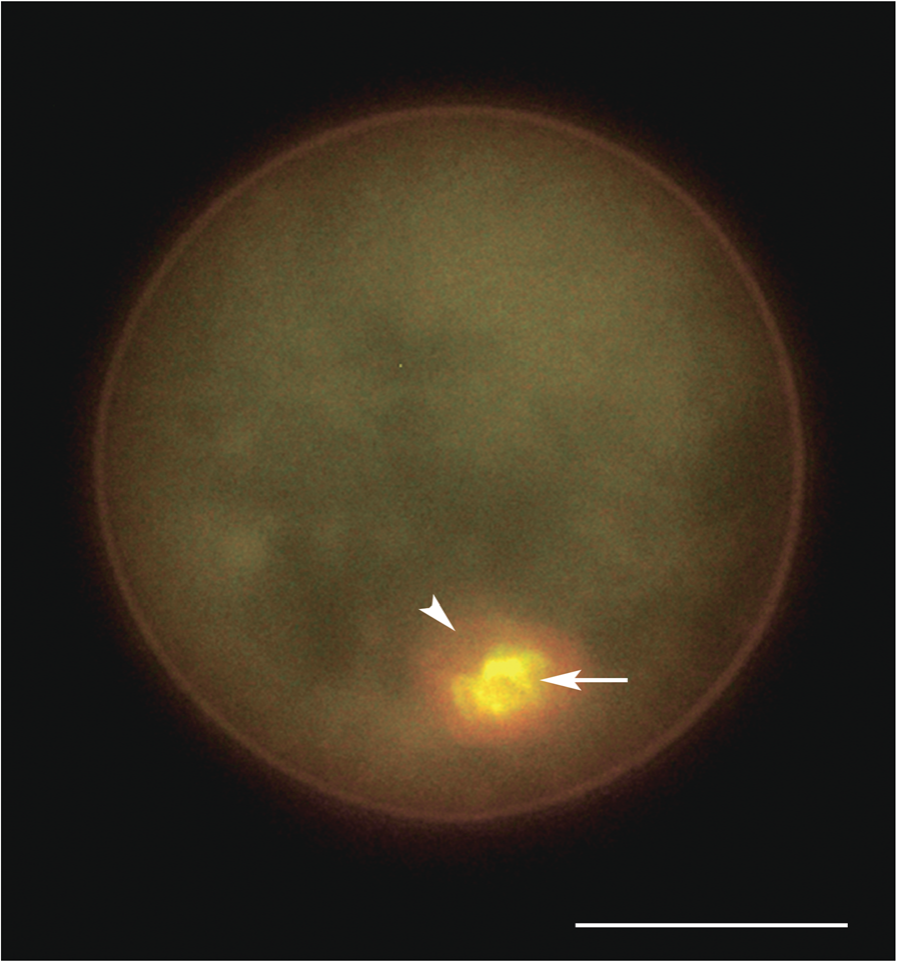
*T. castaneum* GV isolated from vitellogenic (stage VII) oocyte and stained with acridine orange. Note that all DNA is concentrated in the karyosphere (arrow); the karyosphere capsule (arrowhead) is highly enriched in RNA which is also unevenly distributed in the nucleoplasm. Bar represents 20 μm

Fluorescent staining of *T. castaneum* GVs with acridine orange showed that all DNA is concentrated in the karyosphere, as expected. At the same time, RNA is unevenly distributed in the nucleoplasm and highly enriches in the KC that contains the vast majority of oocyte RNA (Fig. 6). The nature of this RNA is not known. We would like to establish whether the KC is an RNA storage place or active transcription occurs at the vicinity of the KC.

Using BrUTP assay, we found that *T. castaneum* oocyte chromosomes united into the karyosphere maintain residual transcription even in the late stage VII oocytes, predominately in the PR that contains chromatin loops and perichromatin fibrils. The hyperphosphorylated form of RNA polymerase II and basal transcription factors (TFIID and TFIIH) as well as poly(A)^+^ RNA have also been demonstrable in the PR of the *T. castaneum* karyosphere. The residual transcription in the karyosphere apparently requires not only transcription factors but also RNA processing factors, including the components of the exon junction complex (EJC). The EJC core protein Y14 in the *T. castaneum* KC was revealed with antibodies [8]. In the present study, we employed a gene engineering approach to have a dynamic picture of the distribution of the newly synthesized protein in the GV. The Myc-tagged Y14 plasmid-based construct is the template (Fig. 1c, b) for the capped mRNA to be injected into the oocyte.

### Y14–Myc RNA microinjections in *T. castaneum* oocytes

**Fig. 7:**
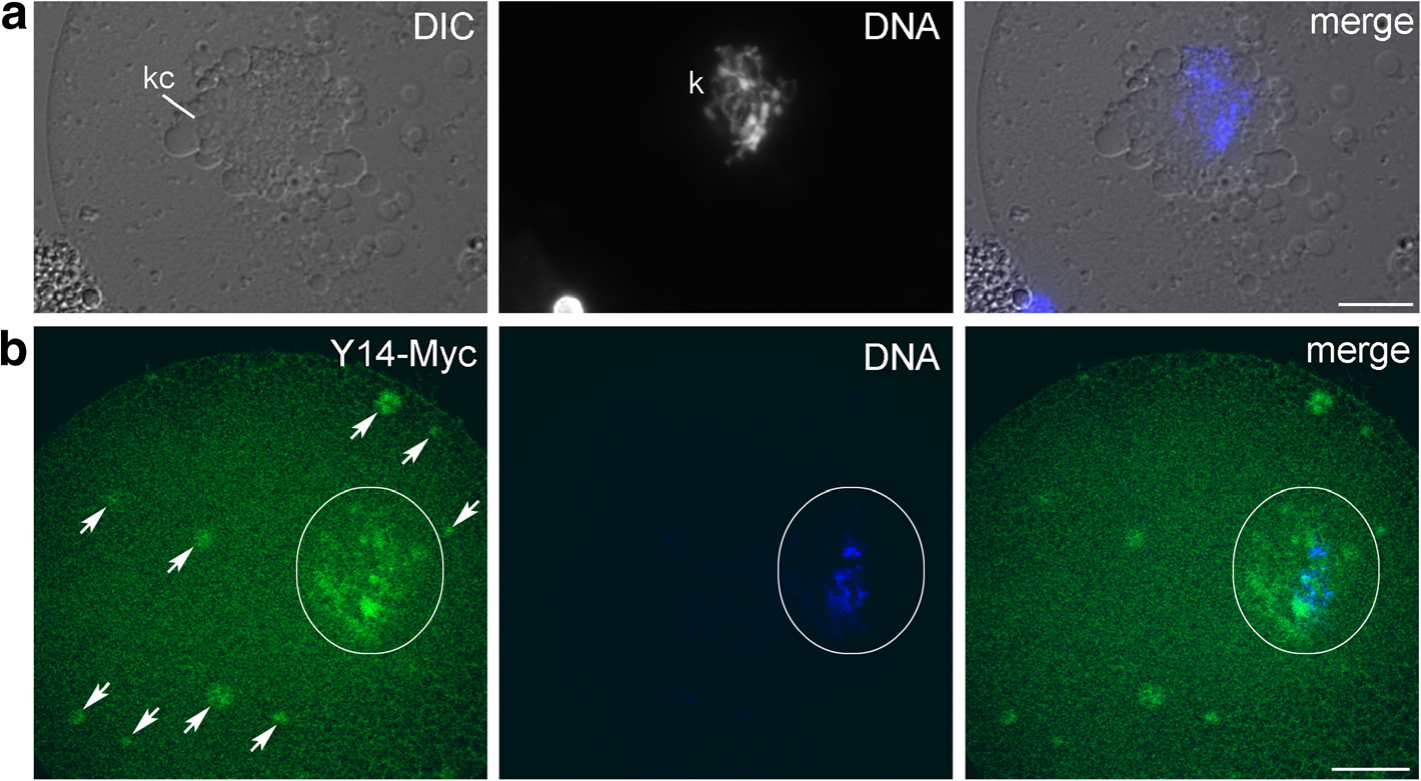
Squashed *T. castaneum* GVs (stage V) after microinjections of Y14-Myc mRNA into the ooplasm. **a**, DIC with DAPI staining; karyosphere capsule (kc) and karyosphere (k) are marked. **b**, Staining with anti-Myc AB (*green*) and anti-DNA dye To-Pro-3 (*blue*); nuclear area containing the karyosphere and its capsule is encircled; *arrows* indicate nucleoplasmic nuclear bodies. Bars represent 10 μm

**Fig. 8:**
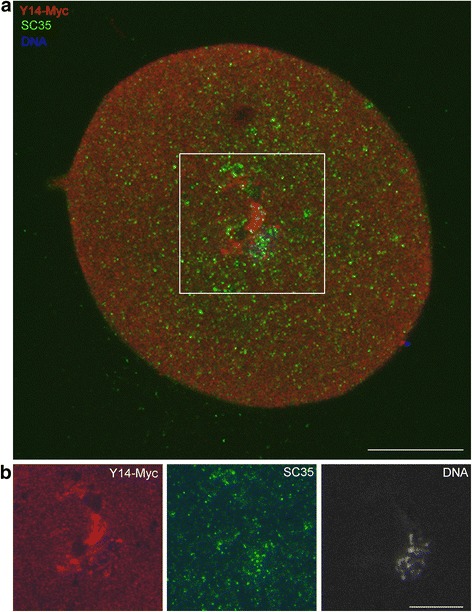
*T. castaneum* GV isolated from vitellogenic (stage VII) oocyte microinjected with Y14–Myc mRNA transcribed in vitro. **a**, The merged image of the whole GV. **b**, Localization of Y14–Myc (*red*) in the area outlined in A; SC35 (*green*); DNA was counterstained with To-Pro-3 (grey scale) to reveal the karyosphere. Bars represent 10 μm

Injection into insect oocyte is not an easy task by itself and counted as impossible until BrUTP was successfully injected [48]. Here, we used late previtellogenic (stage V) and vitellogenic (stage VII) oocytes with, respectively, partially or fully formed KC in microinjection experiments. 5′-end capped Y14-Myc RNA was synthesized on the template of the 639 bp fragment from the plasmid B (Fig. 2a, II). This RNA was injected into the ooplasm. The karyosphere with the KC and nuclear bodies are well-recognizable using DIC optics (Fig. 7a). During 3–5 h following mRNA injection, translation of the fusion protein actively occurs, and the protein successfully traveled into the GV (Fig. 7b). In the stage V oocyte (late previtellogenesis), Y14–Myc was localized in the KC. Nucleoplasmic nuclear bodies were also found enriched in the fusion protein at this stage (Fig. 7b). In later oocytes (the stage VII, vitellogenesis), the fusion protein was also revealed in the vicinity of condensed chromatin, in the KC, but not in the vast majority of SC35 domains (speckles) (Fig. 8). At higher magnification (Fig. 8b), some DNA is seen to extend from the condensed chromatin in the karyosphere into the PR. Decondensed chromatin in the PR of the karyosphere can be clearly detected at the ultrastructural level with the use of anti-DNA antibody (Fig. 5b). Myc-positive foci were also observed in the nucleoplasm, but they did not coincide with SC35 domains as revealed by double immunostaining with Myc antibodies (Fig. 8).

**Fig. 9:**
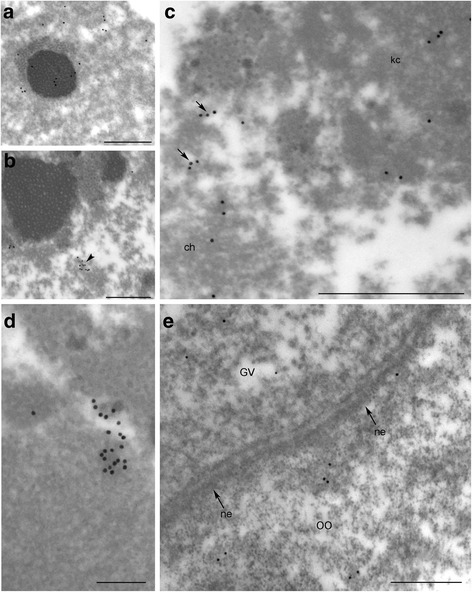
Ultrathin sections of *T. castaneum* GVs from oocytes injected with Y14–Myc mRNA after labeling with anti-Myc AB. **a**, Labeled nuclear body (SC35 domain) in the stage V oocyte. **b**, Similar nuclear body devoid of labels in the stage VII oocyte; a patch of labels (*arrowhead*) is visible and does not belong to the body. **c**, An overview of the fragment of karyosphere (ch, chromatin) and its capsule (kc); labels masking the fibrillar material in the perichromatin region are marked by arrows. **d**, The fragment of the karyosphere capsule; a patch of labels does not correspond SC35 domain. **e**, Anti-Y14–Myc labels near the nuclear envelope (ne); GV, germinal vesicle (oocyte nucleus); oo, ooplasm. Bars represent 0.5 μm in **a**—**c**, **e** and 200 nm in **d**

The IEM study confirmed IF data on the distribution and dynamics of Y14–Myc in the *T. castaneum* GV. In the stage V (late previtellogenic) oocytes microinjected with Y14–Myc mRNA, nucleoplasmic nuclear bodies of the 2nd type according to our nomenclature were labeled with antibody against Myc (Fig. 9a), whereas similar domains were devoid of labels in the stage VII (vitellogenic) oocytes (Fig. 9b). We have previously determined the *T. castaneum* oocyte nuclear bodies of the 2nd type as SC35-containing domains [8]. A complex KC is maintained in the injected oocytes, and some anti-Myc labels were observed in the PR of the karyosphere and in a fibrous material composing the capsule (Fig. 9c, d). The design of our experiments allowed reaching equilibrium between Y14–Myc synthesized in the ooplasm and the protein moving into the nucleus due to its NLS. Some labels masking the fusion protein was localized at the ultrastructural level close to the nuclear envelope (Fig. 9e).

Y14–Myc mRNA injections into oocyte shows the dynamic pattern of the fusion protein distribution. At the previtellogenic stage, there are two main locations for the protein: SC35 domains (IGCs/speckles) and the KC. At the vitellogenic stage, IGCs/speckles were devoid of the labels, and Y14–Myc was found in the PR of the karyosphere. The latter area apparently includes the places of residual transcription.

## Discussion

Here, we performed a gene engineering-based study to localize Y14 protein in oocyte nuclear structures of a model insect, *T. castaneum*. The oocyte nucleus (GV) of this beetle is characterized by the presence of a meiosis-specific structure formed by condensed chromosomes (the karyosphere), and of the extrachromosomal structures including the karyosphere capsule (KC) and various nuclear bodies. Since the *T. castaneum* karyosphere retains residual transcription activity [8], it was not surprising that Y14 involved in the processes of gene expression localizes to the perichromatin region (PR) of the karyosphere in all developmental stages studied. Our finding that this protein demonstrates a dynamic pattern of intranuclear distribution in respect of specific structures such as the KC and nuclear bodies is more essential. While in previtellogenic oocytes Y14 localizes to both the KC and SC35-containing bodies analogous to nuclear speckles/IGCs, a destination site for this protein in late vitellogenic oocytes is the KC only. Our results accentuate the specificity of oocyte nuclear structures and suggest that the KC as a peculiar nuclear compartment of oocytes may be involved in nuclear metabolism of RNAs and proteins rather than representing just a passive barrier to separate the condensed chromosomes assembled into a karyosphere from the rest of the nucleoplasm.

### RNA content and residual transcription in the karyosphere

The *T. castaneum* GV staining for total RNA (Fig. 6) has shown that late vitellogenic oocyte GVs contain a significant amount of RNA. The main portion of this RNA accumulates in the area corresponding to the KC. IF and IEM studies unexpectedly revealed that the KC in the oocytes of *T. castaneum* [8] and also of another beetle, *Anthonomus pomorum* [14], is highly enriched in small nuclear (sn) RNPs. In particular, snRNAs revealed in the KC of these species carry the 2,2,7-trimethylguanosine (TMG) cap, characteristic of the 5′ end of many splicing snRNAs that have already completed the cytoplasmic period of their processing [49]. Interestingly, the “Sm-epitope” of snRNPs was detected recently in the KC of frog oocytes [50] (Ilicheva et al., 2016). Even in the species without a well-developed fibrous capsule (e.g., *Tenebrio molitor* or *Panorpa communis*), oocyte chromosomes are tightly associated with a fibrogranular material containing snRNPs and other RNA transcription and processing factors [51–53].

The presence of RNA in the KC is in agreement with the idea that this nuclear domain represents a specialized part of oocyte nuclear matrix [12, 13]. In somatic cells, nuclear matrix in vivo contains RNPs and a set of proteins associated with RNA together with the structural proteins [54, 55].

Certain structural proteins (F-actin, lamin B) are prominent compounds of the *T. castaneum* KC [8]. F-actin was revealed as a basic component of the KC in several insects [56, 57] and also in frogs [58]. It has been shown that actin plays a crucial role in the maintenance of oocyte nuclear architectonics, and its depolymerization leads to a collapse of nuclear structures [59]. It is likely, that nuclear actin can form complexes with Y14, as well as with mRNA export adapter Aly/REF and export receptor NXF1, as revealed with the use of FRET analysis carried out in mouse early embryos [60]. Also, the active role of nuclear actin in transcription [61, 62] and chromatin remodeling [63] is well-documented for somatic cells.

It cannot be excluded that a KC develops when the karyosphere maintains residual transcriptional activity in late oocytes. In tenebrionid beetles, a karyosphere usually has an external KC [64] with the exception of *Tenebrio* [52, 53]. BrUTP incorporation assay showed that chromatin in the karyosphere devoid of a capsule (the scorpionfly, *Panorpa*, and the yellow mealworm, *Tenebrio*) is transcriptionally silent at the final stages of oocyte growth [48], whereas the transcriptional activity in oocytes with a well-developed KC does not entirely ceased. In such the organisms as the lacewing, *Chrysopa* [65], the darkling beetle, *Blaps* [66], and the grass frog, *Rana temporaria* [67], which possess a KC, residual RNA synthesis in the GV was found. In contrast, incorporation of ^3^H–uridine was not registered in a human karyosphere (karyosome) which is devoid of a capsule [68]. The question which RNA is last-synthesized in the oocyte with rather inactivated genome has a principal significance for developmental biology. It could be expected that RNAs deposited in a KC, especially those last-synthesized during oogenesis, might be essential for the organization of future embryo.

In the *T. castaneum* GV of the stage V–VII oocytes, the sites of residual transcription are clearly defined, representing the PR of the karyosphere. Y14-Myc targeted to this area after microinjection of Myc-tagged Y14 mRNA (Figs. 7b, 8, and 9c). The concept of the PR implies that this area cannot be considered separately from chromatin [69]. It serves not only as the main site of transcribed DNA and nascent pre-mRNAs, but co-transcriptional pre-mRNA processing including splicing, DNA replication and, perhaps, reparation also occur there [70–72]. The KC that separates late transcription in the GV could be a useful model to find out what is the nature of these RNA.

The retention of mRNAs encoding the proteins of the maternal effect is well-documented for the ooplasm and has a significant impact on embryonic development. For instance, Oskar mRNA determines the future primary axes in unfertilized eggs, and Y14–Magoh is responsible to Oskar localization [73]. On the contrary, data on the distribution of different RNA types in the GV are scanty in literature [74, 75]. The organisms with a well-developed KC may be talkative objects in order to solve this issue.

### EJC core protein Y14

The protein to trace in the current work, Y14, is a pre-mRNA splicing factor and EJC core protein. In the eukaryotic cell, mRNA biogenesis intimately depends on the EJC, which had received wide attention due to both its original assembly pathway on mRNAs and the ability to communicate with various mRNA-related cellular machineries [76, 77]. The EJC is loaded onto mRNA by spliceosomes during splicing at a precise point upstream the exon-exon junctions and serves as a binding platform for mRNA export factors [78, 79]. Y14 is one of the best-characterized proteins of the EJC core. This core is able to interact directly with pre-mRNA and is assembled prior to exon ligation [80]. At this stage, Y14 associates with pre-mRNA [81, 82] and accompanies the spliced mRNA until translation in the cytoplasm [83, 84]. A special domain YNS/NLS located near the N-terminus of the Y14 molecule is simultaneously serves as a NLS and also confers Y14 nuclear export [85]. Also, Y14 contains a well-defined RNA recognition motif (RRM, Additional file 1: Figure S1), but it is buried almost entirely at the interface of the Y14–Magoh heterodimer [86–88]. This suggests that Y14 does not bind pre-mRNA directly, and this binding requires one or more intermediary proteins. Nevertheless, Y14 is found associated with both nuclear and newly exported cytoplasmic mRNAs.

The approach based on oocyte microinjections with synthesized in vitro Myc-tagged Y14 mRNA allowed us to trace nuclear dynamics of the newly synthesized protein. We found that this RNA microinjected in oocytes is being translated successfully for 3 h, and the product of translation is imported into the GV. A significant level of fluorescence was registered in the PR of the karyosphere where loose DNA loops are located. Y14 is localized at different oocyte stages in a different way. In the stage V oocytes, at the beginning of KC formation, it is revealed in SC35 domains; however, Y14 enriches a well-formed KC rather than SC35 domains at later stages. It could be supposed that like in somatic cells [16] oocyte SC35-containing nuclear bodies represent transient domains for some proteins involved in mRNA transport and export. It is well-known that somatic extrachromosomal nuclear domains contain not only resident slow-mobile proteins including the proteins of the nuclear matrix. For instance, pre-mRNA splicing factors located in IGCs/speckles are highly dynamic and are being stored in IGCs not longer than in other parts of the nucleoplasm [89] in spite of their 5–10 times higher concentration in IGCs [90]. It is possible that oocyte nuclear bodies may contain other proteins that are included as compounds of the KC at the end of oocyte growth; Myc-Y14 fusion protein is the first which dynamic distribution was confirmed.

Chromatin rearrangement, which is a characteristic feature of oocyte maturation, is well documented; we showed that it accompanied with the movement of a nuclear protein in the case of residual transcription during genome inactivation.

## Conclusion

Our data presented here suggest the KC is not only a specialized part of nuclear matrix supporting the karyosphere (a knot of condensed chromosomes) and separating it from the products of chromosome activity, as believed previously. Using a gene engineering approach with injections of mRNA derived from the Myc-tagged Y14 plasmid-based construct, we showed for the first time that the KC is a destination site for a nuclear protein involved in mRNA splicing and export. Thus, we believe that the KC could represent a special nuclear compartment involved in the processes of gene expression in the case the karyosphere retains residual transcription activity. However, it is still unclear, why the KC appears during oogenesis in some species and does not exist in closely related others, which exhibit the same structural and functional pattern of the ovaries and possess similar biological features. Further comparative studies using a complex of modern approaches, including in vivo studies, are required.

## Additional files

Additional file 1: Figure S1. Protein alignment of *Tribolium castaneum* Y14 (XP_967777), *Drosophila melanogaster* Y14 (NP_610454) and *Homo sapiens* Y14 (NP_005096). Y14 RNA binding/recognition domain (RRM/RBM8) indicated with *grey*. Identical amino acids highlighted in *black*, similar in *grey*. Alignment and visualization performed with Geneious 6 Software (TIFF 181 kb)

Additional file 2: Figure S2. Differences between Y14 splicing systems in human and *T. castaneum* cells. A, The genome sequence of *T. castaneum* Y14. B, Normally spliced Y14 mRNA of *T. castaneum*. C, Abnormal splicing of *T. castaneum* Y14 mRNA in human HEK293 cells (TIFF 204 kb) Green, exons; blue, introns.

## Abbreviations

EJC: Exon-exon junction complex
GV: Germinal vesicle (oocyte nucleus)
IEM: Immunoelectron microscopy
IF: Immunofluorescence
IGC: Interchromatin granule cluster
KC: Karyosphere capsule
PR: Perichromatin region
