# Supplementary figures and images for: The exon junction complex factor Y14 is dynamic in the nucleus of the beetle Tribolium castaneum during late oogenesis
Source: Mol Cytogenet. 2017 Nov 9;10:41. doi: 10.1186/s13039-017-0342-4 (PMC5679382; doi:10.1186/s13039-017-0342-4)

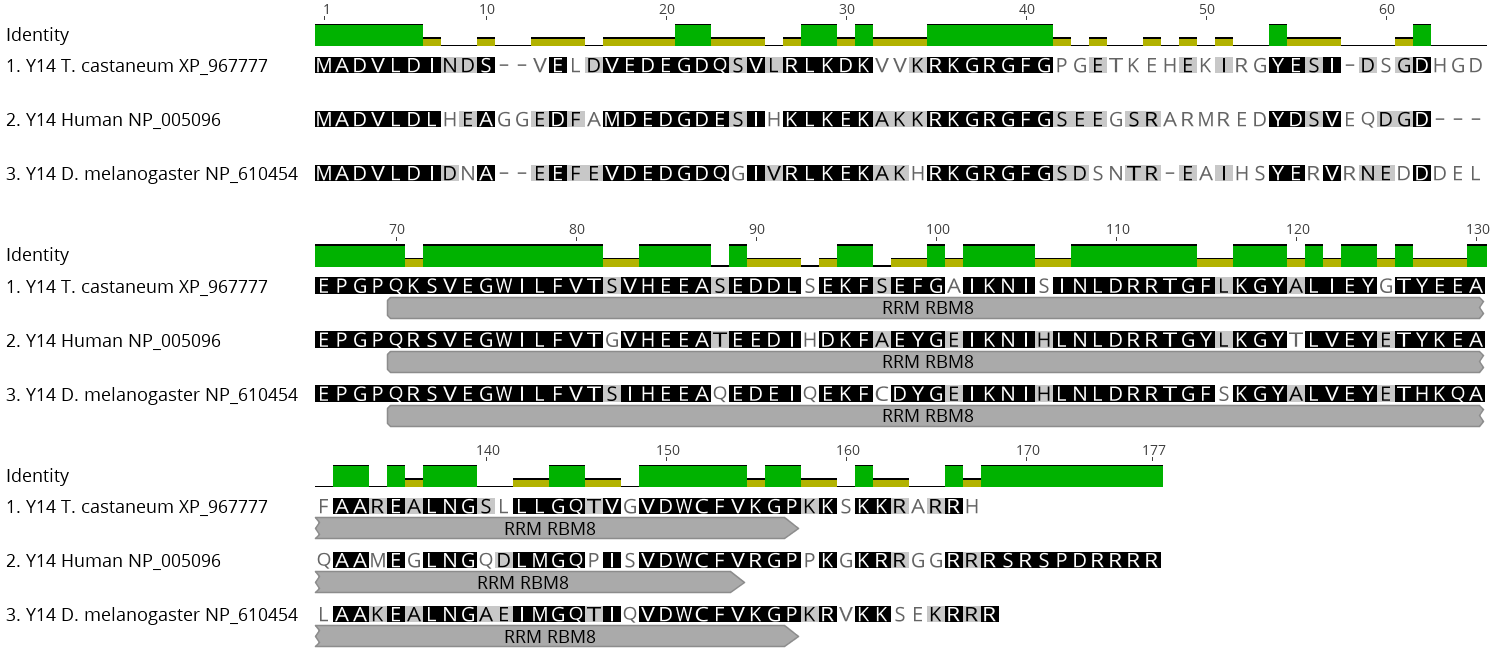

Supplement: Supplementary file 1 — Protein alignment of Tribolium castaneum Y14 (XP_967777), Drosophila melanogaster Y14 (NP_610454) and Homo sapiens Y14 (NP_005096). Y14 RNA binding/recognition domain (RRM/RBM8) indicated with grey. Identical amino acids highlighted in black, similar in grey. Alignment and visualization performed with Geneious 6 Software (TIFF 181 kb) [file 13039_2017_342_MOESM1_ESM.tif]

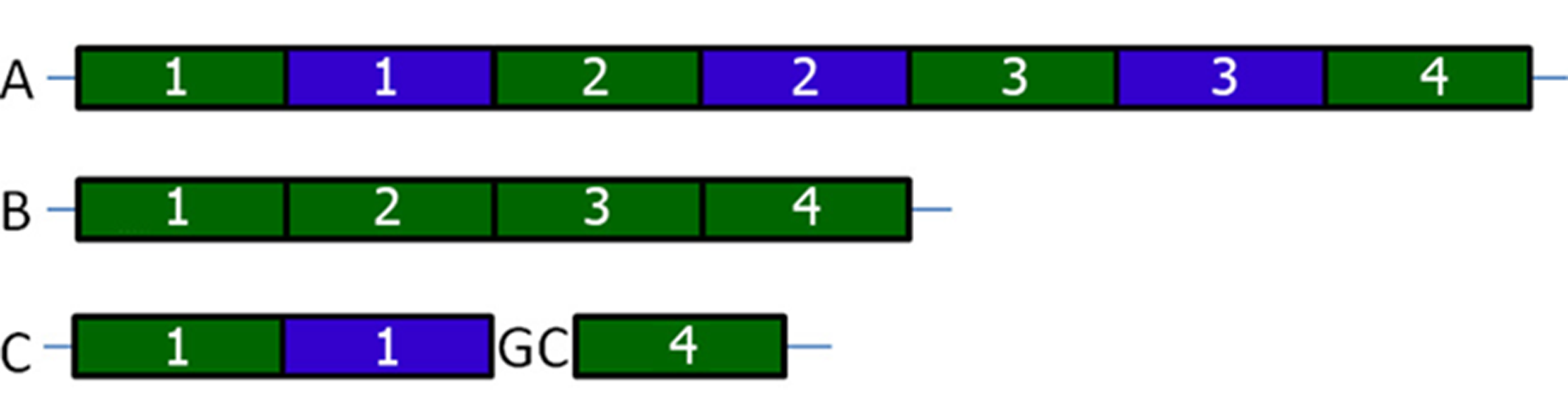

Supplement: Supplementary file 2 — Differences between Y14 splicing systems in human and T. castaneum cells. A, The genome sequence of T. castaneum Y14. B, Normally spliced Y14 mRNA of T. castaneum. C, Abnormal splicing of T. castaneum Y14 mRNA in human HEK293 cells (TIFF 204 kb) Green, exons; blue, introns. [file 13039_2017_342_MOESM2_ESM.tif]
